# Measuring the Impact of Medical Science Liaisons: A Global Cross-Sectional Survey of Evaluation Practices and Perceptions

**DOI:** 10.7759/cureus.105099

**Published:** 2026-03-12

**Authors:** Samuel J Dyer, Cherie Hyder, Jeff Kraemer, Paul Ward, Larry Dollar

**Affiliations:** 1 Medicine, Medical Science Liaison Society, Miami, USA; 2 Medical Affairs, Immunocore, Radnor, USA; 3 Medical Affairs, Medical Science Liaison Society, Miami, USA; 4 Medical Affairs, BeOne Medicines, Cambridge, USA; 5 Medical Affairs, Syneos Health, Bridgewater, USA

**Keywords:** health care professional (hcp), key opinion leader (kol), key performance indicators (kpis), medical affairs, medical science liaison (msl), pharmaceutical

## Abstract

Introduction: Medical science liaisons (MSLs) serve a critical role in pharmaceutical, biotechnology, medical device, diagnostics, and healthcare companies by facilitating scientific exchange with key opinion leaders (KOLs) and health care professionals (HCPs). Despite their growing strategic importance, evaluating MSL impact remains challenging due to the complexity of their responsibilities. This study investigates current practices for assessing MSL impact, explores how their value is defined, and examines the challenges in measuring MSL performance.

Methods: A cross-sectional online survey was conducted, targeting MSLs, MSL managers/directors, MSL Excellence and Operations, executive management, and other medical affairs professionals. Participants were recruited via professional networks, social media, pharmaceutical industry conferences, online platforms dedicated to medical affairs, and posting on LinkedIn, resulting in a non-probability convenience sample. The survey comprised multiple-choice and Likert-scale questions covering KOL engagement impact scores, definitions of MSL value and impact, and challenges in performance, measurement, and assessment.

Results: A total of 1023 respondents participated. Geographically, the majority of respondents were based in the United States (56%), followed by Spain (6%), Brazil (5%), and Canada (5%), with additional contributions from Europe, Latin America, and other regions. Overall, 52% indicated that KOL engagement impact scores should be utilized as a performance metric. MSL value was most often defined as building and maintaining strong KOL relationships (27%), whereas MSL impact was most frequently linked to influencing clinical practice and improving patient care (46%). Key challenges included difficulty defining and measuring the quality of MSL activities (76%) and reliance on quantitative standards that do not reflect MSLs' value (61%). Only 40% reported that their organizations measure MSL impact, and 41% suggested that the assessment should combine individual achievements with team contributions. Furthermore, 67% considered measuring MSL performance "difficult" or "very difficult".

Conclusion: This study highlights the challenges of measuring MSLs' impact and value. Many organizations continue to struggle with capturing the quality of MSL activities and their broader strategic contributions.

## Introduction

Medical science liaisons (MSLs) serve an integral, strategic function within the medical affairs departments of pharmaceutical, biotechnology, medical device, diagnostics, and other healthcare companies [1,2]. Their primary responsibilities include providing accurate, evidence-based information to key opinion leaders (KOLs) and other health care professionals (HCPs), supporting clinical decision-making, and contributing to improved patient outcomes. In contrast to sales representatives, MSLs function in a non-commercial, scientific role and, consequently, are not incentivized by sales targets or market share expansion. However, accurately measuring and demonstrating the impact of MSL activities remains a significant challenge for many organizations.

Most organizations prioritize quantitative key performance indicators (KPIs) such as the number of KOL or HCP interactions, field days, presentations, time spent in the field, and attendance at scientific meetings [1,3,4]. While quantitative KPIs provide measurable data, they primarily reflect activity volume and often fail to capture the broader value that MSLs provide to both internal and external stakeholders, such as influencing clinical decision-making, facilitating knowledge transfer, fostering cross-functional collaboration, and driving evidence generation [5,6]. These strategic contributions are more effectively evaluated through qualitative methods, including HCP feedback, the quality of insights captured, and documented examples of strategic impact. However, qualitative KPIs are frequently underutilized due to their inherent subjectivity and the challenges of standardizing measurements [7,8]. Although KOL and HCP feedback surveys can be used to evaluate MSL performance, they often provide limited insight due to high costs and low response rates, making it difficult to draw broader conclusions about MSL impact.

The healthcare landscape is rapidly evolving, driven by advancements in personalized medicine, artificial intelligence (AI), and telemedicine, the widespread dissemination of medical information through social media, and the growing complexity of treatment [4-6,9]. This evolution has significantly expanded the responsibilities of MSLs, making their roles increasingly multifaceted and strategically focused [2]. In particular, AI is emerging as a transformative technology that is reshaping how MSLs operate by facilitating data-driven insights, enabling predictive analytics, and enhancing the precision of KOL engagement strategies [9]. Although the original primary purpose of MSLs was to facilitate the exchange of scientific information with KOLs, the role has expanded significantly. Today, MSLs are responsible for gathering actionable insights and engaging a broad range of external stakeholders, including KOLs, HCPs, digital opinion leaders (DOLs), patient advocacy groups, pharmacists, study site personnel, and clinical researchers. In addition, MSLs also collaborate closely with internal stakeholders and cross-functional teams, including sales, marketing, health economics and outcomes research (HEOR), market access, and regulatory affairs, among others. Increasingly, MSLs are expected to align their scientific activities with broader organizational objectives, assist with evidence generation, and support clinical decision-making. Beyond their role in scientific exchange with KOLs and other HCPs, MSLs advance evidence-based decision-making within their organizations by gathering actionable insights, facilitating cross-functional collaboration, supporting data-driven planning and education efforts, and contributing to the medical strategy for the products they support, ultimately with the goal of improving patient outcomes [10,11]. This evolving scope of responsibilities highlights the inadequacy of traditional activity-based metrics and reinforces the need for a hybrid approach that incorporates both quantitative and qualitative performance measures. Research supports the use of this hybrid approach to more accurately assess the strategic value, scientific influence, and overall impact of MSLs, capturing aspects of performance that quantitative metrics alone often fail to measure [5,6,8,11-13]. Effectively measuring and communicating the impact of MSLs is essential for demonstrating the strategic contribution of field medical affairs teams. This ensures that MSL efforts are recognized both internally and externally and provides tangible evidence of their contributions to clinical decision-making and medical strategies, as well as to aligning field insights with organizational objectives.

This study examines current practices and methods for defining, measuring, and evaluating the impact and value of MSLs, identifies key challenges, and outlines preferred approaches to performance assessment. Specifically, this research explores the use of KOL engagement impact scores, definitions of MSL value, definitions of MSL impact, challenges in measuring MSL performance, MSL impact measurement, preferred methods for measuring impact, and perceived difficulty in measuring MSL performance. The findings may influence how organizations assess and optimize the value MSLs provide to their organization and to the KOLs and HCPs they support.

## Materials and methods

Study design and participants

A cross-sectional survey was conducted by the Medical Science Liaison Society to examine the current practices for measuring MSL impact. The survey targeted individuals in medical affairs, including MSLs, MSL managers and directors, MSL Excellence and Operations, executive leadership, and other roles involved in evaluating the impact of MSLs. The study design has been described in detail in a previous publication [13].

The inclusion criteria required the respondents to work in medical affairs or field-based scientific exchange roles. Specifically, participants were required to answer "yes" to the following question: Are you currently employed as an MSL (including medical advisors or other equivalent titles), do you currently manage MSLs, or do you currently work in MSL Excellence/Operations? The only exclusion criterion was answering "no" to this question. Therefore, all participants were required to currently be employed as an MSL, as an MSL leader, or in an MSL Excellence/Operations role.

The objective of this survey was to gather insights from a global perspective, ensuring broad representation. Participants were recruited through professional networks, social media, pharmaceutical industry conferences, and online platforms dedicated to medical affairs. All responses were collected anonymously to encourage honesty and candid feedback.

The survey was posted on LinkedIn to reach participants, resulting in a non-probability convenience sample; therefore, response rates could not be tracked or calculated. No information regarding dropouts or incomplete responses is available because only complete surveys were tracked and calculated.

Survey design

The survey was developed based on a comprehensive literature review and consultations with MSLs and MSL leaders. The survey questions consisted of a mix of multiple-choice and Likert-scale items. Survey data were compiled and analyzed using descriptive statistics. For all questions, the results are presented as the percentage of respondents selecting each option. To enhance clarity and relevance, the survey was pretested with a small sample of MSLs and medical affairs leaders before being finalized.

The final survey was conducted between March 15, 2024, and May 28, 2024, and was designed to address seven key topics related to MSL impact and performance assessment. These topics included the use of KOL engagement impact scores, which evaluate composite metrics used to assess MSL effectiveness in engaging KOLs, how organizations define the value of MSLs, how organizations define the impact of MSLs, the primary challenges associated with measuring MSL performance and value, whether organizations currently measure MSL impact, the methods that may be used to measure MSL impact, and the perceived difficulty of accurately measuring MSL performance.

Data analysis

Data were collected and analyzed using Alchemer survey software (version 6.9.1) (Denver, CO, USA). Descriptive statistical methods were applied to summarize the quantitative responses using frequency distributions and percentages. All responses, including those from MSLs, MSL managers, and other medical affairs professionals, were analyzed collectively without subgroup segmentation, as the objective was to provide a comprehensive overview rather than role-specific comparisons. The results are summarized in 10 tables, each highlighting a key aspect of MSL performance measurement and presenting participant demographics.

Ethics approval and consent to participate

This study involved a voluntary, anonymous global survey of medical affairs professionals. No patient data, personally identifiable information (PII), or sensitive health information was collected. As such, the research did not require ethics committee approval under prevailing international guidelines (e.g., Declaration of Helsinki, Council for International Organizations of Medical Sciences (CIOMS), and US Common Rule). All respondents were informed of the survey's purpose, and informed consent was implied through voluntary participation.

## Results

A total of 1023 medical affairs professionals from 63 countries participated in the global survey. Participant characteristics are presented below. The majority of the respondents (55%) were MSLs, senior MSLs, or medical advisors (or equivalent titles). Management roles, including managers and directors of MSLs, accounted for 26%, while executive management or vice presidents of medical affairs, along with those in MSL Excellence or Operations, each comprised 8%. Only 3% of participants were categorized as "Other" (Table 1).

**Table 1:** Current role. MSL: medical science liaisons

What is your current role?	
MSL/Sr. MSL/medical advisor (or equivalent title)	55%
Manager/director of MSLs (or equivalent title)	26%
Executive management/vice president of medical affairs	8%
MSL Excellence/Operations	8%
Other	3%

The survey included responses from a wide variety of company types. A total of 40% of participants worked for large pharmaceutical/biotechnology companies. Medium-sized pharmaceutical/biotechnology companies accounted for 25% of respondents, while small pharmaceutical/biotechnology companies accounted for 23%. The remaining 12% were distributed among medical devices (5%), diagnostic companies (3%), contract MSL organizations (2%), contract research organizations (1%), and other (1%) (Table 2).

**Table 2:** Company type. MSL: medical science liaisons; CRO: contract research organization

How would you classify your company?	
Large pharmaceutical/biotechnology (revenue of USD 10+ billion)	40%
Medium pharmaceutical/biotechnology (revenue of USD 1-10 billion)	25%
Small pharmaceutical/biotechnology (revenue of less than USD 1 billion)	23%
Medical device	5%
Diagnostic company	3%
Contract MSL organization	2%
CRO	1%
Other	1%

Geographically, the majority of respondents were based in the United States (56%), followed by Spain (6%), Brazil (5%), and Canada (5%). Additional notable representation came from Germany (3%), the United Kingdom (3%), Australia (3%), France (2%), Italy (2%), and Mexico (2%). Although participation levels varied, responses were received from medical affairs professionals in more than 60 countries, reflecting a broad and globally diverse sample. Many other countries accounted for less than 1% of the respondents, and the dataset was primarily concentrated in North America and Europe (Table 3).

**Table 3:** Geographical location.

In which country do you work?					
Country	Percentage	Country	Percentage	Country	Percentage
Algeria	<1%	Greece	<1%	Poland	<1%
Argentina	<1%	Hong Kong	<1%	Portugal	1%
Australia	3%	India	1%	Romania	<1%
Austria	<1%	Indonesia	<1%	Russia	<1%
Bangladesh	<1%	Iraq	<1%	Serbia	<1%
Belgium	<1%	Ireland	<1%	Singapore	1%
Brazil	5%	Israel	<1%	Slovakia	<1%
Canada	5%	Italy	2%	South Africa	<1%
Cape Verde	<1%	Jamaica	<1%	South Korea	1%
Chile	<1%	Japan	1%	Spain	6%
China	<1%	Jordan	<1%	Sweden	<1%
Colombia	<1%	Malaysia	<1%	Switzerland	1%
Costa Rica	<1%	Mexico	2%	Taiwan	<1%
Cote d'Ivoire	<1%	Morocco	<1%	Thailand	<1%
Croatia	<1%	Netherlands	<1%	Turkey	<1%
Czech Republic	<1%	New Zealand	<1%	Ukraine	<1%
Denmark	<1%	Nigeria	<1%	United Arab Emirates	<1%
Dominican Republic	<1%	Norway	<1%	United Kingdom	3%
Egypt	<1%	Pakistan	1%	United States	56%
France	2%	Paraguay	<1%	Uruguay	<1%
Germany	3%	Peru	<1%	Venezuela	<1%

When asked whether KOL engagement impact scores should be utilized to measure MSL performance, 52% of respondents agreed. However, 30% indicated that they were not sure, and 19% disagreed with using this metric in their organizations (Table 4).

**Table 4:** KOL engagement impact scores. KOL: key opinion leader; MSL: medical science liaisons

KOL engagement impact scores, which is a composite metric that quantitatively assesses the effectiveness and influence of interactions between MSLs and KOLs, should be utilized in your organization to measure MSL performance	
True	52%
False	19%
I am not sure	30%

When defining the value of MSLs, 27% of respondents indicated their ability to build and maintain strong relationships with KOLs. Another 24% indicated that the definition of value is subjective and often determined by internal leadership's expectations of what MSLs should contribute to company success. A further 19% defined MSL value based on the quality and depth of scientific knowledge and expertise they provide, while 13% identified their effectiveness in educating HCPs about therapeutic areas and treatment options. A total of 8% referenced the impact MSLs have on patient outcomes and healthcare practices, and 6% recognized their contributions to clinical research and the generation of insights that inform drug development and strategy. An additional 4% selected "Other" (Table 5). 

**Table 5:** ​​​​​​​Defining the value of an MSL. MSL: medical science liaisons; KOLs: key opinion leaders; HCP: health care professionals

How do you define the value of an MSL within your organization?	
By their ability to build and maintain strong relationships with KOLs	27%
It's subjective based on what internal leadership thinks MSLs should be doing to contribute to company success	24%
Through the quality and depth of scientific knowledge and expertise they provide	19%
By their effectiveness in educating HCP about therapeutic areas and treatment options	13%
Through their impact on patient outcomes and healthcare practices	8%
Through their contribution to clinical research and insights that inform drug development and strategy	6%
Other	4%

When evaluating the impact of an MSL within their organization, 46% of participants indicated that the impact was defined by the influence on clinical practice, behavioral changes that enhance patient care, or outcomes. Another 22% defined impact by the number and quality of KOL engagements and relationships established, while 12% chose the level of contribution to scientific and medical knowledge within the organization. An additional 11% selected the effectiveness in addressing and resolving scientific and medical inquiries from HCPs, and 5% defined MSL impact as the support provided in advancing clinical trials and research initiatives. Another 5% of participants selected "Other" (Table 6).

**Table 6:** Definition of MSL impact. KOL: key opinion leader; MSL: medical science liaisons

How do you define the impact of an MSL within your organization?	
Through the influence on clinical practice, behavioral changes that enhance patient care, or outcomes	46%
By the number and quality of KOL engagements and relationships established	22%
By the level of contribution to scientific and medical knowledge within the organization	12%
By the effectiveness in addressing and resolving scientific and medical inquiries from health care professionals	11%
Through the support provided in advancing clinical trials and research initiatives	5%
Other	5%

The primary challenges in measuring the performance and value of MSLs include difficulty in defining and measuring the quality of MSL activities (76%) and performance standards, such as quantitative measures, that do not reflect the value MSLs provide (61%). Challenges related to the variability of access to KOLs/HCPs across different regions/territories, as well as demonstrating the impact and value of MSLs within the organization, were each selected by 53% of participants. The short-term focus of some KPIs that do not capture long-term value was identified as a challenge by 51% of respondents. Other challenges selected included the need for compliant measures that reflect MSL value and impact (44%), the lack of standardized metrics across the industry (43%), the variability in MSL roles and responsibilities across different therapeutic areas (38%), insufficient tools or systems to capture and analyze relevant data (36%), and the communication gap between MSLs and management regarding expectations (28%). Only 3% selected "Other" (Table 7).

**Table 7:** Challenges in measuring MSL performance. MSL: medical science liaisons; KOLs: key opinion leaders; HCP: health care professionals; KPIs: key performance indicators

What are the primary challenges in measuring the performance and value of MSLs? (Select all that apply)	
Difficulty defining and measuring the quality of MSL activities (KOL engagements, internal projects, etc.)	76%
Performance standards such as quantitative measures do not reflect the value MSLs provide	61%
Variability of access to KOLs/HCPs across different regions/territories	53%
Demonstrating the impact and value of MSLs within the organization	53%
Short-term focus of some KPIs not capturing long-term value	51%
Need for compliant measures that reflect MSL value and impact	44%
Lack of standardized metrics across the industry	43%
Variability in MSL roles and responsibilities across different therapeutic areas	38%
Insufficient tools or systems to capture and analyze relevant data	36%
The communication gap between MSLs and management regarding expectations	28%
Other	3%

When asked if their organizations measure MSL impact, responses were split: 40% selected "Yes," 39% selected "No," and 21% selected "I am not sure" (Table 8).

**Table 8:** Measuring MSL impact. MSL: medical science liaisons

Does your organization measure the impact of MSLs?	
Yes	40%
No	39%
I am not sure	21%

Regarding how the impact of MSLs should be measured within organizations, 41% of respondents selected through the combined contribution to the company's overall targets. Slightly fewer (40%) indicated it should be measured by the results of the collaborative team efforts they are involved in. Only 15% selected through the direct results they achieve individually, while 4% selected "Other" (Table 9). 

**Table 9:** Approaches to measuring MSL impact. MSL: medical science liaisons

How should the impact of MSLs be measured within your organization?	
Through the combined contribution to the company's overall targets	41%
By the results of collaborative team efforts they are involved in	40%
Through the direct results they achieve individually	15%
Other	4%

Finally, the majority of respondents rated the difficulty of accurately measuring MSL performance as either "Difficult" (51%) or "Very difficult" (16%), while 27% chose "Neutral," 6% chose "Easy," and only 1% selected "Very easy" (Table 10).

**Table 10:** Difficulty in measuring MSL performance. MSL: medical science liaisons

How would you rate the difficulty of accurately measuring the performance of MSLs within your organization?	
Very difficult	16%
Difficult	51%
Neutral	27%
Easy	6%
Very easy	1%

## Discussion

The increasing complexity of the MSL role in pharmaceutical and other healthcare companies necessitates an evaluation of their value and impact, as well as how these are assessed. This survey investigated current practices in defining the impact of MSL performance, the challenges involved, and how MSLs and medical affairs professionals perceive these issues.

While the majority of respondents (52%) supported using KOL engagement impact scores as composite metrics to measure MSL impact, 30% were unsure. Overall, the survey results indicate that there is still a lack of consensus across organizations regarding the most effective method(s) to assess MSL performance. This uncertainty emphasizes the need for more structured, evidence-based evaluation frameworks.

When evaluating MSL performance, one of the most frequently cited challenges was the difficulty of measuring the quality of their activities (27%). This aligns with the fact that only 40% of organizations assess the impact of MSLs and a high percentage of respondents (67%) find it difficult or very difficult to measure. These findings highlight a recurring concern in medical affairs: the value and impact of the scientific exchange facilitated by MSLs are difficult to quantify and measure.

Previous research supports the results of this study, demonstrating that traditional performance metrics often fail to capture the contributions of MSLs, especially qualitative metrics such as relationship-building and scientific dialogue [1,8,14]. There is a growing recognition of the need to move beyond metrics that simply count interactions. Instead, organizations should evaluate the depth, quality, and strategic relevance of MSL engagements. This may include using composite indicators that reflect both qualitative insights and alignment with broader medical objectives rather than relying primarily on basic activity counts [8,15]. Digital platforms and emerging technologies, including AI-powered analytics, may help to capture these nuanced indicators in a scalable and compliant manner.

Regarding the definition of MSL value, 27% of respondents indicated that it is based on their ability to build and maintain strong relationships with KOLs. This aligns with previous research identifying KOL trust and relationship strength as the core of MSL effectiveness [16]. Respondents also defined MSL value by the quality and depth of scientific knowledge and expertise they provide (19%), their effectiveness in educating HCPs about therapeutic areas and treatment options (13%), their impact on patient outcomes and healthcare practices (8%), and their contribution to clinical research and insights that inform drug development and strategy (6%). These varied definitions reflect the broader issues highlighted in prior studies, such as a lack of standardization and high variability in role expectations, which may affect performance evaluation, retention, career progression, and alignment with organizational goals [4-6,8].

When participants were asked how they defined the impact of an MSL within their organization, 46% indicated that it should be related to their influence on clinical practice, behavioral changes that enhance patient care, or outcomes. This reflects an evolving shift in perception, transitioning from the traditional view of MSLs as primarily providers of scientific information to recognizing them as active contributors to healthcare quality and clinical decision-making [2,4,5,7,8,17]. This trend highlights the necessity of aligning MSL activities and performance metrics directly with the medical strategy of the products they support.

Finally, 41% of the respondents indicated that the impact of MSLs should be measured through the combined contribution of collaborative team efforts and individual performance, with only 15% preferring measurements based on the direct results they achieve individually. These findings reinforce previous studies, which emphasize that MSL performance may best be evaluated through team-based and organizational outcomes, acknowledging the inherently collaborative and cross-functional nature of their role [1,4-7].

Based on the survey findings, several actions to address the gaps in defining, measuring, and demonstrating the impact and value of MSLs are recommended. Organizations should establish a clear definition of MSL value across the organization to ensure consistency in how MSL activities and contributions align with the medical strategy for the product they support. Simultaneously, organizations should develop a standardized evaluation of the impact of MSLs, emphasizing how MSL activities support KOL clinical decision-making and advance strategic objectives.

The establishment of standardized and consistent processes to track and assess the impact of MSLs is further recommended, ensuring that these evaluations are directly aligned with broader strategic goals and outcomes and account for the varying levels of impact of different MSL activities. These results suggest that assessing MSL value should be standardized within organizations to maintain alignment with both the medical and strategic objectives of the supported products.

This study had several limitations. First, the survey relied on self-reported data, which may have introduced response bias, as participants' perceptions may not always accurately reflect objective practices. In addition, despite the robust sample size, the possibility of self-selection bias cannot be dismissed. Furthermore, this analysis did not account for variations in responses based on factors such as company type, participant role (i.e., MSL, MSL leader, etc.), therapeutic area, or geographical region, which may influence perspectives and should be explored in future research. All responses were analyzed in aggregate, regardless of the respondent's role or company type.

Future studies should focus on identifying more effective methods to measure the impact of MSLs across various therapeutic areas and the life-cycle stages of products. Both MSL value and impact assessments require refinement to accurately reflect the full scope of MSL contributions, particularly concerning clinical outcomes and alignment with product strategies. Research should also investigate the obstacles to effective performance evaluation, including the challenges associated with assessing collaborative teamwork. The exploration of real-world data, machine learning, and digital tools for evaluating MSL performance and impact should be prioritized. Ultimately, studies that provide insights into the evolution of the MSL profession and the necessary adaptations in measurement practices are essential and would offer a significant contribution to research in this area, where information remains limited.

## Conclusions

This study illustrates the complex challenges in defining, measuring, and demonstrating the impact of MSLs. Although MSLs are recognized for their critical role in disseminating accurate, evidence-based information to KOLs and other HCPs, which supports clinical decision-making, there remains a significant need for more accurate, standardized, and strategic approaches to assess MSL contributions. By addressing the gaps identified in this research and adopting specific strategies for evaluating the impact of MSLs, organizations can more effectively demonstrate their strategic value and optimize their contributions to medical objectives.

Organizations should adopt enhanced evaluation frameworks that incorporate both team-based outcomes and qualitative contributions. These frameworks must capture the full scope of the value that MSLs deliver, not only to the company but also to the KOLs they support and ultimately to patients. To be effective, performance measurement should be closely aligned with the medical strategy of the product and be capable of measuring the MSL's impact on KOL and HCP engagement and scientific education, as well as their contribution to more informed clinical decision-making.
